# Genome Sequence of *Serratia marcescens* MSU97, a Plant-Associated Bacterium That Makes Multiple Antibiotics

**DOI:** 10.1128/genomeA.01752-16

**Published:** 2017-03-02

**Authors:** Miguel A. Matilla, Zulema Udaondo, Tino Krell, George P. C. Salmond

**Affiliations:** a Department of Biochemistry, University of Cambridge, Cambridge, United Kingdom; b Department of Environmental Protection, Estación Experimental del Zaidín, Consejo Superior de Investigaciones Científicas, Granada, Spain; c Abengoa Research, Biotechnology Technological Area, Seville, Spain

## Abstract

*Serratia marcescens* MSU97 was isolated from the Guayana region of Venezuela due to its ability to suppress plant-pathogenic oomycetes. Here, we report the genome sequence of MSU97, which produces various antibiotics, including the bacterial acetyl-coenzyme A (acetyl-CoA) carboxylase inhibitor andrimid, the chlorinated macrolide oocydin A, and the red linear tripyrrole antibiotic prodigiosin.

## GENOME ANNOUNCEMENT

Bacteria belonging to the *Serratia* genus are ubiquitous in the environment, and some *Serratia marcescens* strains are associated with nosocomial infections (1, 2). However, numerous plant-associated *S. marcescens* strains have been reported, some of which were shown to promote plant growth through the synthesis of phytohormones, secretion of exoenzymes, production of siderophores and bioactive molecules, or by the induction of systemic resistance (3–6).

*Serratia marcescens* MSU97 was isolated from the stems of a native aquatic plant (*Rhyncholacis pedicillata*) that grows in the Carrao River of the Venezuelan Guayana (4). MSU97 was the most abundant isolated bacterium found in healthy *R. pedicillata* plants, and this plant protection phenotype was associated with its ability to inhibit the growth of plant-pathogenic oomycetes, fungi, and bacteria (4, 7, 8). Thus, the strain synthesizes various secondary metabolites, including the antibacterial compound andrimid (8), the antifungal and antioomycete haterumalide oocydin A (7, 9), and the red tripyrrole antibiotic prodigiosin (4). MSU97 was also shown to be highly virulent in *Caenorhabditis elegans* infection models (7). We have reported recently that another plant-associated *Serratia* strain produces zeamine, a hybrid polyketide-nonribosomal peptide with nematicidal activity (10), but the virulence mechanism(s) of MSU97 is currently unknown. MSU97 produces quorum-sensing signaling molecules (9), and the strain was used as a model bacterium for the investigation of the biosynthesis and regulation of oocydin A and andrimid (7–9).

The genomic DNA of MSU97 was purified using the DNeasy blood and tissue kit (Qiagen) and *de novo* sequenced at the Department of Biochemistry (University of Cambridge, United Kingdom) using 454 DNA pyrosequencing technology on a Pico Titer plate for a Roche Applied Science Genome Sequencer FLX system. The resulting 521,156 reads (204 Mb of raw data) were *de novo* assembled using Newbler version 2.6, resulting in an approximately 38× coverage of the estimated genome size. This assembly resulted in 65 contigs larger than 1,000 bp. The largest contig was 418,649 bp, and the average contig size was 77,365 bp. The genome was automatically annotated using the NCBI Prokaryotic Genome Annotation Pipeline version 3.0 (http://www.ncbi.nlm.nih.gov/genome/annotation_prok).

The assembled genome of MSU97 includes 5,258,534 bp, with an overall G+C content of 57.8%. Automated genome annotation predicted 4,652 protein-coding sequences, 45 pseudogenes, one regularly interspaced short palindromic repeat (CRISPR) array, six rRNAs (5S, 16S, and 23S), 70 tRNA genes, and nine noncoding RNAs. In addition to the biosynthetic clusters responsible for the production of andrimid, oocydin A, and prodigiosin, *in silico* analyses using antiSMASH (11) predicted five additional gene clusters presumed to be involved in the synthesis of nonribosomal peptides and polyketides. These results highlight the potential of *Serratia* strains as an extraordinary and underexploited source of bioactive secondary metabolites (12). Further analyses of the genome sequence may enable the identification of new genes putatively involved in plant growth promotion, in addition to providing more insight into the biosynthesis and regulation of structurally diverse secondary metabolites.

### Accession number(s).

The sequences obtained by this whole-genome shotgun project have been deposited in DDBJ/EMBL/GenBank under the accession number MJAO00000000.
